# Tracing Human IgE B Cell Antigen Receptor-Bearing Cells With a Monoclonal Anti-Human IgE Antibody That Specifically Recognizes Non-Receptor-Bound IgE

**DOI:** 10.3389/fimmu.2021.803236

**Published:** 2021-12-20

**Authors:** Mohammed Zghaebi, Maria Byazrova, Sabine Flicker, Sergio Villazala-Merino, Nicholas J. Campion, Victoria Stanek, Aldine Tu, Heimo Breiteneder, Alexander Filatov, Musa Khaitov, Verena Niederberger-Leppin, Julia Eckl-Dorna, Rudolf Valenta

**Affiliations:** ^1^Department of Otorhinolaryngology, Medical University of Vienna, Vienna, Austria; ^2^National Research Centre (NRC) Institute of Immunology, Federal Medical-Biological Agency (FMBA) of Russia, Moscow, Russia; ^3^Department of Immunology, Faculty of Biology, Lomonosov Moscow State University, Moscow, Russia; ^4^Division of Immunopathology, Department of Pathophysiology and Allergy Research, Center for Pathophysiology, Infectiology and Immunology, Medical University of Vienna, Vienna, Austria; ^5^Division of Medical Biotechnology, Department of Pathophysiology and Allergy Research, Center for Pathophysiology, Infectiology and Immunology, Medical University of Vienna, Vienna, Austria; ^6^Immunology Department, Pirogov Russian National Research Medical University, Moscow, Russia; ^7^Department of Clinical Immunology and Allergy, Sechenov First Moscow State Medical University, Moscow, Russia; ^8^Karl Landsteiner University of Health Sciences, Krems, Austria

**Keywords:** omalizumab, IgE, B cells, allergy, CD23, spiking, PBMCs, FcεRI

## Abstract

Up to 30% of the population suffers from immunoglobulin E (IgE)-mediated allergies. Despite current stepwise gating approaches, the unambiguous identification of human IgE-producing cells by flow cytometry and immunohistology remains challenging. This is mainly due to the scarcity of these cells and the fact that IgE is not only expressed in a membrane-bound form on the surface of IgE-producing cells in form of the B cell antigen receptor (BCR), but is more frequently found on various cell types bound to the low and high affinity receptors, CD23 and FcϵRI, respectively. Here we sought to develop a sequential gating strategy for unambiguous detection of cells bearing the IgE BCR on their surface. To that aim we first tested the monoclonal anti-IgE antibody omalizumab for its ability to discriminate between IgE BCR and receptor-bound IgE using cells producing IgE or bearing IgE bound to CD23 as well as basophils exhibiting FcϵRI receptor-bound IgE. Using flow cytometry, we demonstrated that omalizumab recognized IgE producing cells with a high sensitivity of up to 1 IgE^+^ cell in 1000 human peripheral blood mononuclear cells (PBMCs). These results were confirmed by confocal microscopy both in cell suspensions as well as in nasal polyp tissue sections. Finally, we established a consecutive gating strategy allowing the clear identification of class-switched, allergen-specific IgE^+^ memory B cells and plasmablasts/plasma cells in human PBMCs. Birch pollen specific IgE^+^ memory B cells represented on average 0.734% of total CD19^+^ B cells in allergic patients after allergen exposure. Thus, we developed a new protocol for exclusive staining of non-receptor bound allergen-specific IgE^+^ B cell subsets in human samples.

## Introduction

Allergy, a worldwide disease affecting up to 30% of the world population, is characterized by immunoglobulin E (IgE) production specific to the culprit allergens (1). Though IgE is continuously produced and returns to baseline levels within few days after removal by extracorporeal immunoadsorption in sensitized patients (2), the location and the extent of contribution of IgE B cell antigen receptor (BCR) bearing memory B cells (MBCs) to human IgE production is not fully clarified (3, 4). This is mainly due to limited knowledge of these cells (5) as the characterization of human IgE-producing cells in blood by flow cytometry is challenging due to several reasons: Firstly, IgE BCR bearing cells are extremely rare in the blood. They are estimated to represent between 0.0019 and 0.3% of total B cells in allergic subjects (6–9) and to contribute to 0.2% of the human serum IgE (4, 10). Secondly, the IgE BCR is expressed at much lower levels than BCRs composed of other immunoglobulins such as IgG or IgM (7). This might be due to the suboptimal polyadenylation signals in the IgE transcripts (11), which makes the clear distinction of these cells from background staining more difficult. Thirdly and most importantly, IgE occurs in two different forms on the surface of immune cells: in the form of an IgE BCR (12, 13) or bound to its high or low affinity receptors, FcϵRI and CD23 respectively. In addition, other IgE binding factors have been described such as epsilon-binding protein (14). FcϵRI is mainly present on the surface of basophils, mast cells and dendritic cells, while CD23 is predominantly expressed by B cells and monocytes (15, 16). Thus, especially CD23-bound IgE renders the detection of IgE^+^ BCR bearing cells difficult as many commonly used anti-human IgE antibodies are unable to discriminate between the membrane-expressed and receptor-bound form of IgE.

To exclude B cells bearing IgE bound to CD23 from the analysis of IgE^+^ B cells, various approaches have been tried. Early strategies included stripping IgE from CD23 by lactic acid wash (17, 18). However, this treatment may be damaging for the cells especially if they are planned to be used further on e.g. for functional assays after flow cytometric sorting (19). The compromise of excluding cells double positive for IgE and CD23 in the flow cytometer comprises the danger of accidentally removing true IgE^+^ B cells having both free as well as CD23-bound IgE (20). Thus, more recent approaches to circumvent this issue applied stepwise gating for IgE^+^ memory B cells and plasmablasts (PBs)/plasma cells (PCs) firstly using anti-CD19 and anti-CD38, followed by sequential exclusion of IgM^+^, IgD^+^, IgA^+^ and IgG^+^ cells (6, 7, 21) or intracellular staining for IgE for identification of IgE-producing cells (5, 8). Nevertheless, these strategies identify IgE^+^ cells only indirectly by stepwise exclusion of other cells and may also miss IgE^+^ B cells having IgG bound to their FcγRIIb receptor (22–24). Therefore, a flow cytometric approach allowing for the direct and clear identification of IgE^+^ BCR bearing and IgE-producing B cells in human samples is needed.

Several anti-IgE antibodies have been developed for the treatment of severe forms of allergy (20). Among these, omalizumab is the only one licensed for treatment. It reduces the levels of free IgE in the blood by 99% within a few hours after administration (25) and is successfully used to treat severe asthma and urticaria (26, 27). It is known to bind to the Cϵ3 region of IgE where the contact sites for both CD23 and FcϵRI are situated. As a result, omalizumab is thought to bind to IgE only in its free and not in its receptor-bound form and is thus a candidate for exclusively detecting IgE^+^ BCR bearing MBCs or IgE^+^ PBs/PCs (28).

In this study we investigated the suitability of fluorescently labelled omalizumab for the staining of non-receptor bound IgE^+^ B cells by flow cytometry. Furthermore, we aimed to establish a consecutive gating strategy for the clear identification of allergen-specific IgE^+^ BCR bearing MBCs and PBs/PCs based on double staining with omalizumab and allergen.

## Material and Methods

### Cell Lines

A human Epstein Barr virus-transformed B-cell line (EBV B cells) expressing CD23 (15) and the human IgE myeloma cell line (U266) (29) were cultured in Roswell Park Memorial Institute (RPMI) 1640 Glutamax medium (ThermoFisher Scientific, Waltham, Massachusetts) supplemented with 10% fetal bovine serum (ThermoFisher Scientific) and 1% penicillin/streptomycin (ThermoFisher Scientific) at 37°C and 5% CO_2_. Cell counting was performed in the presence of Trypan blue to ensure a cell viability of more than 80%. CD23 expression on the surface of EBV B cells was confirmed by flow cytometry (see **Supplementary Table S1**).

### Protein Expression (Bet v 1) and Purification

A codon-optimized synthetic gene encoding Bet v 1.0101 was inserted into the pET28b(+) expression vector (Novagen, Darmstadt, Germany) and expressed in *Escherichia coli* BL21[DE3] cells (New England Biolabs, MA, USA). Expression was carried out in standard *E. coli* culture media at 30°C. Protein expression was induced with 1 mM isopropyl β-D-1-thiogalactopyranoside at an OD600 nm of 0.8. After overnight expression, cells were harvested, cell pellets were frozen for 24 hours, and then lysed by freeze-thawing 3 times in 50 mM sodium phosphate buffer, pH 6.0, supplemented with protease inhibitor tablets (Roche Diagnostics, Basel, Switzerland) and 10 mM dithiothreitol. Purification was achieved *via* hydrophobic interaction chromatography (Phenyl Sepharose 6 Fast Flow, GE Healthcare, Uppsala, Sweden), ion exchange chromatography (Q Sepharose Fast Flow, GE Healthcare) and gel chromatography filtration on a Sephacryl S-200 HR column (GE Healthcare). The purified Bet v 1 protein was dialyzed against 10 mM sodium phosphate buffer, pH 7.5. Endotoxin Removal Beads (Miltenyi Biotec, Gladenbach, Germany) were used to remove lipopolysaccharides. The protein was stored at – 80°C until use.

### Peripheral Blood Mononuclear Cells (PBMCs) Preparation and Basophil Isolation

Heparinized blood was obtained from birch pollen allergic patients (n=5) and non-allergic subjects (n=3) after obtaining a written informed consent. Blood drawing was performed with the approval of the Ethics Committee of the Medical University of Vienna (EK508/2011). Where indicated, patients had been provoked with birch pollen extract on three consecutive days starting one week prior to the blood draw (EK1320/2021).

PBMCS were isolated from heparinized human blood using Ficoll density gradient centrifugation (GE Healthcare). Enrichment of basophils was performed using the MACS Basophil Isolation Kit II (Miltenyi Biotech) according to the manufacturer’s protocol and as previously described (30). The purity of the isolated basophils was confirmed by staining for CD123 and FcϵRI by flow cytometry (see **Supplementary Table S1**).

### Flow Cytometry

The anti-human IgE antibody omalizumab (=Xolair ^®^, Novartis, Basel, Switzerland) as well as human IgG1,κ (Sigmaaldrich, St. Louis, Missouri) were labelled with Alexa Fluor 647 Conjugation Kit-Lightning-Link (Abcam, Cambridge, MA) and Bet v 1 was labelled with PE/R-Phycoerythrin Conjugation Kit Lightning-Link (Abcam) according to the manufacturer’s instructions. For the surface staining of PBMCs, basophils, and EBV B cells, cells were incubated for 20 minutes on ice in blocking buffer of 1% bovine serum albumin (BSA), 5% goat serum, 5% mouse serum in PBS (Morphisto, Frankfurt am Main, Germany). Thereafter, EBV B cells were incubated for another 20 minutes in the aforementioned blocking buffer on ice with humanized chimeric monoclonal IgE specific for the major birch pollen allergen Bet v 1 (31) at a final concentration of 6 µg/ml. After washing, cells were stained with respective antibodies (see **Supplementary Table S1**) and fixable viability dye e780 (ThermoFisher Scientific) for 20 minutes on ice in PBS containing 1% BSA (Sigmaaldrich). Bet v 1-PE staining was only performed for PBMCs. Cells were then washed 3 times and resuspended in blocking buffer.

For intracellular staining of U266 cells, cells were stained with fixable viability dye e780 (ThermoFisher Scientific) prior to fixation with formalin 4% (Morphisto) at 37°C for 10 minutes. Cells were then washed and incubated with a permeabilization buffer (PBS, 1% BSA, 0.1% Saponin) containing 5% goat serum and 5% mouse serum for 20 minutes. After washing, cells were stained with respective antibodies (see **Supplementary Table S1**) in permeabilization buffer. After staining, cells were rinsed again 3 times with 1% BSA in PBS and resuspended in the same buffer for acquisition in the flow cytometer. For PBMC spiking experiments, U266 cells at indicated numbers were mixed with indicated numbers of PBMCs prior to fixation and intracellular staining as described above for U266 cells.

Compensation beads (ThermoFisher Scientific) were used to optimize acquisition settings. Furthermore, quality control measures to sustain the instrument’s optics, fluidics, and electronics according to the manufacturer’s instructions were performed regularly by using CS&T beads (BD bioscience, Franklin Lakes, New Jersey, USA). Flowjo software version 10.7.2 (Tree Star Inc., Ashland, Oregon, USA) was used to analyze data obtained on a BD LSRFortessa (BD Biosciences). At least 100,000 alive cells were acquired per sample.

### Cell and Tissue Preparation for Confocal Analysis

Single cell suspensions of EBV-transformed B cells, U266 cells and enriched basophils were prepared onto slides using Shandon Cytospin 3 (ThermoFisher Scientific). A nasal polyp sample (EK1956/2018) embedded in OCT medium and stored at -80°C was cut in 5 µm cryosections. Subsequently U266 cells were spiked onto the tissue slide using the cytospin. Thereafter tissue was fixed using 4% formalin (Morphisto) and respective antibodies (see **Supplementary Table S2**) were applied to the cryosections overnight in PBS (Morphisto) containing 0.1% Saponin (Sigmaaldrich) and 2% of bovine serum albumin (BSA). Slides were washed and stained with the secondary antibody for CD19 for 1 hour at room temperature. Slides were counterstained with DAPI (ThermoFisher Scientific) to reveal nuclei and mounted with ProLong Gold Antifade Mountant (ThermoFisher Scientific). Images of slides were acquired using a confocal laser scanning microscope (Nikon, Tokyo, Japan, 60x magnification).

### Statistical Analysis

For the FACs staining of U266 cells, EBV B cells, and basophils, Flowjo-generated frequencies and gated event counts were exported to Excel (Microsoft, Redmond, WA) to calculate the mean and standard deviation for three independent experiments. For the linearity assessment for the spiking of PBMCs with U266, values were calculated as follows:

Percentage of U266 _Observed_ = measured percentage _omalizumab_ – mean measured percentage _isotype_

GraphPad Prism software version 9 (GraphPad Software, La Jolla, CA) was used to calculate Pearson correlation coefficient. A *p* value less than 0.05 was considered statistically significant.

For the quantification of IgE-bearing B cells in the blood of allergen-provoked patients, the percentage of IgE^+^ Bet v 1^+^ MBCs of total B cells was calculated as the percentage of alive CD19^+^ CD38^dim/-^ IgD^-^ IgM^-^ IgE^+^ Bet v 1^+^ cells of total CD19^+^ cells, and the percentage of IgE^+^ Bet v 1^+^ PBs/PCs of total B cells was calculated as the percentage of alive CD19^+^ CD38^+^ IgD^-^ IgM^-^ IgE^+^ Bet v 1^+^ cells of total CD19^+^ cells. The same strategy was applied for the non-allergic individuals.

## Results

### Omalizumab Exclusively Detects-IgE Producing Cells but Not Receptor-Bound IgE in Cellular Flow Cytometric and Confocal Assays

**Figure 1:**
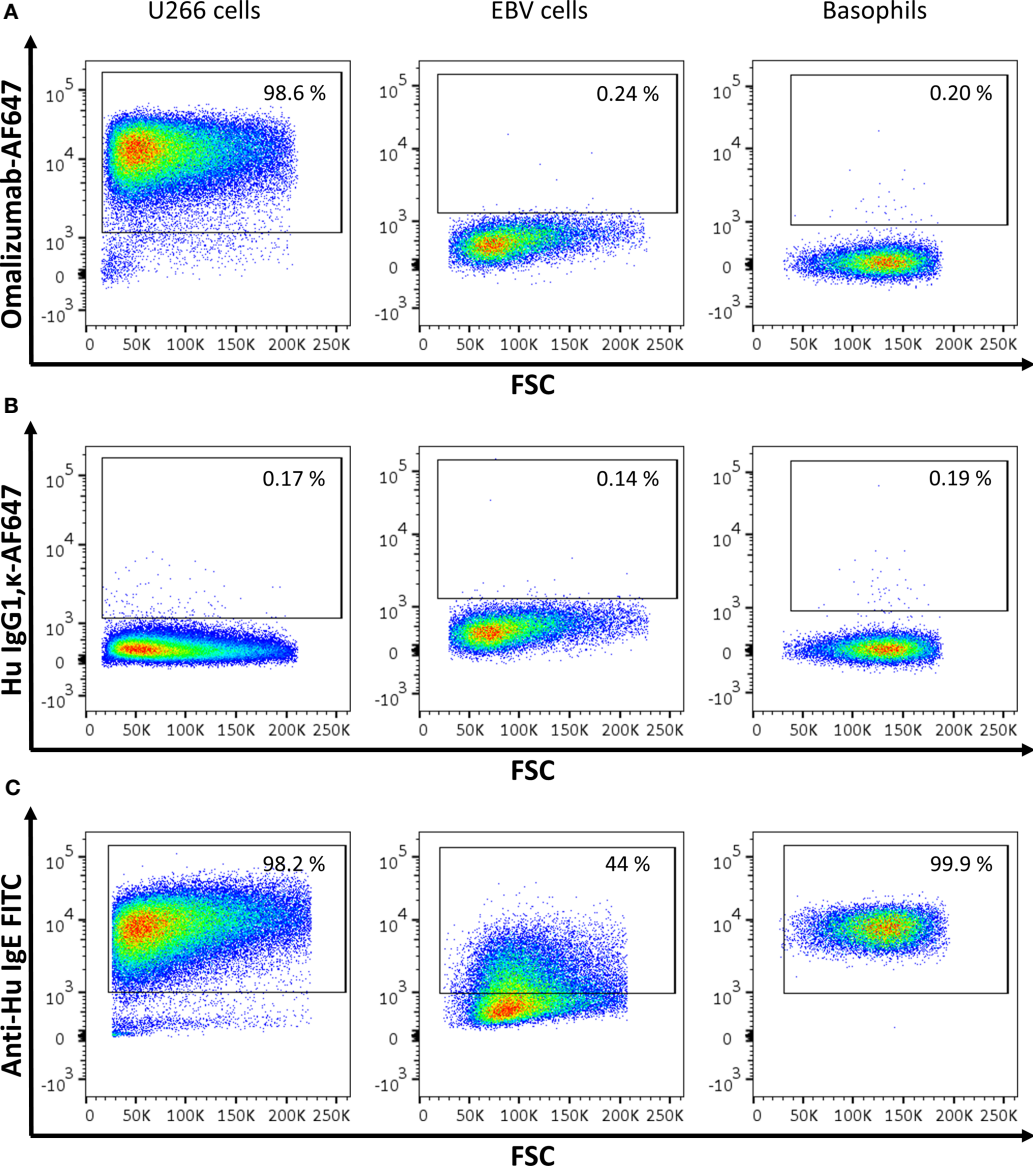
Assessment of IgE-producing cells or cells bearing receptor-bound IgE by omalizumab or polyclonal anti-human IgE using flow cytometry. **(A‒C)** Representative flow cytometric analysis of (left panels) U266, an IgE-producing cell line (U266 cells), (middle panels) an Epstein Barr virus-transformed human cell line bearing IgE bound to the low affinity receptor for IgE, CD23 (EBV cells), and (right panels) basophils enriched from human blood and bearing IgE bound to the high affinity receptor for IgE, FcϵRI. Plots depict forward scatter (x-axis) against (y-axis): **(A)** Alexa Fluor (AF) 647 labelled anti-human IgE antibody omalizumab, **(B)** the respective isotype control (human IgG1, κ) labelled with AF647 and **(C)** polyclonal anti-human IgE FITC. Cells were previously selected for alive cells by negative staining for eFluor 780 viability dye and gates were set using isotype controls for omalizumab-AF647 or unstained cells for anti-human IgE FITC respectively. Experiments were performed in duplicates and representative plots of three independent experiments are shown.

In order to test if the humanized monoclonal anti-human IgE antibody omalizumab fulfilled the requirements of reliably detecting IgE as present in the IgE BCR but not receptor-bound IgE we performed fluorescent staining in three different cell types: (i) In the human IgE myeloma cell line U266, an IgE-producing cell line that has previously been used as a surrogate for BCR-bearing and IgE-producing cells (32, 33) (ii) In an Epstein barr virus (EBV)-transformed B cell line (EBV B cells) expressing high levels of CD23 incubated with humanized birch pollen specific IgE prior to staining (15, 34) and (iii) in human basophils enriched from blood of donors and thus bearing IgE bound to their high affinity receptor FcϵRI. IgE was detected either with omalizumab labelled with AF647, the appropriate isotype control (human IgG1κ labelled with AF647) or a commercially available polyclonal anti-IgE FITC antibody (see **Supplementary Table S1**). Omalizumab-AF647 detected IgE only in U266 cells but was unable to stain IgE if bound to CD23 (EBV cells) or FcϵRI (Basophils) (omalizumab AF-647 positive cells: U266 cells: 96.02 ± 1.89%, EBV cells: 0.20 ± 0.11%, Basophils: 0.41 ± 0.68%). Representative plots of three independent experiments performed in duplicates are shown in **Figure 1A**. No positive staining was observed using the respective isotype control for any of the conditions tested (IgG1κ AF-647 positive cells: U266 cells: 0.71 ± 0.91%, EBV cells: 0.11 ± 0.06%, Basophils: 0.38 ± 0.42%) (**Figure 1B**). IgE-positive cells were observed in all three cell types when polyclonal anti-IgE was used (IgE-FITC positive cells: U266 cells: 96.7 ± 1.24%, EBV cells: 42.87 ± 6.30, Basophils: 91.48 ± 11.24%) (**Figure 1C**).

**Figure 2:**
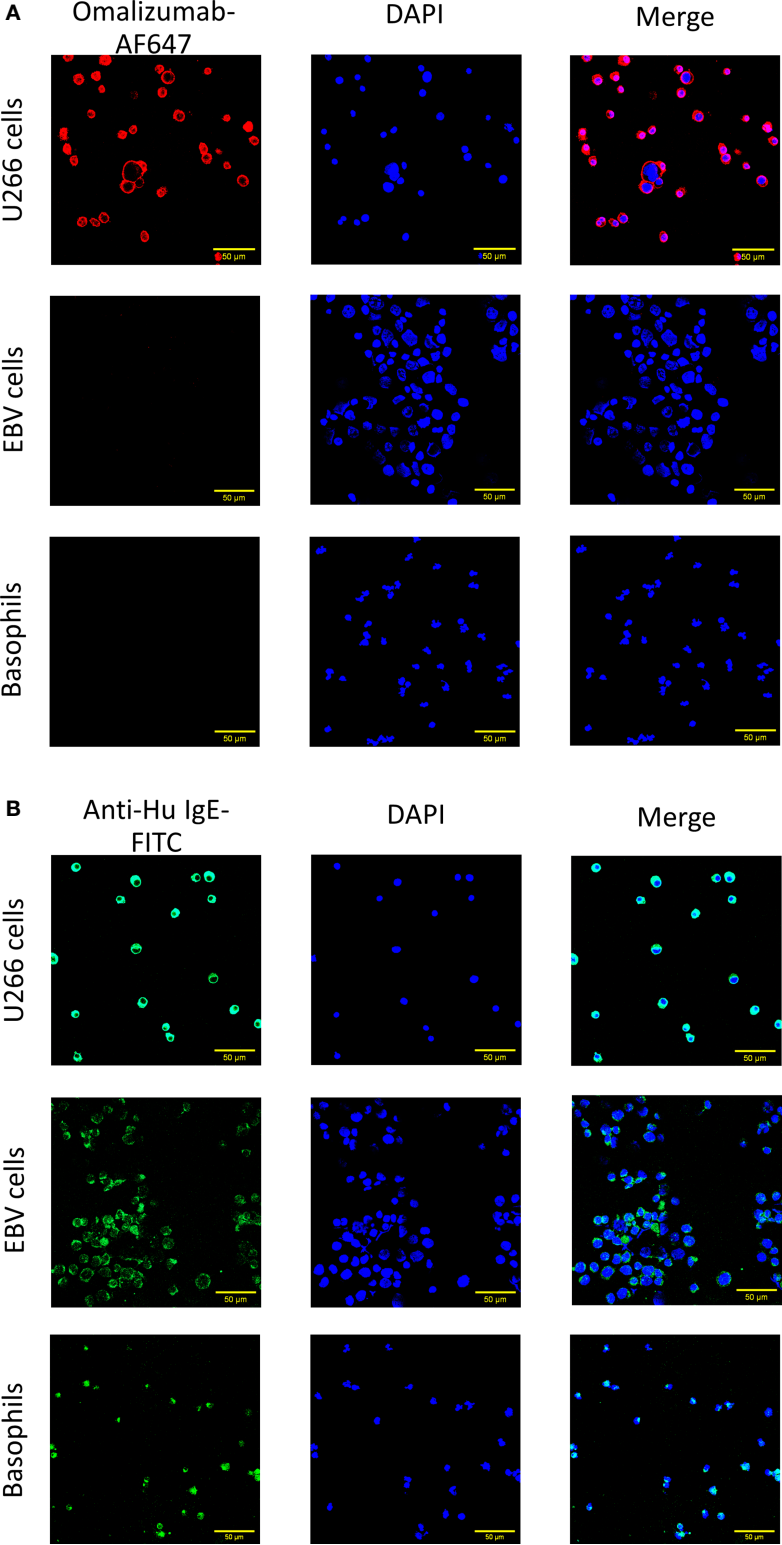
Confocal imaging of IgE-producing cells or cells bearing receptor-bound IgE by omalizumab or polyclonal anti-human IgE. **(A, B)** Representative confocal images of (upper panels) U266, an IgE-producing cell line (U266 cells), (middle panels) an Epstein Barr virus-transformed human cell line bearing IgE bound to the low affinity receptor for IgE, CD23 (EBV cells) and (lower panels) basophils enriched from human blood and bearing IgE bound to the high affinity receptor for IgE, FcϵRI. Cells were stained with **(A)** Alexa Fluor (AF) 647 labelled anti-human IgE antibody omalizumab (red) or **(B)** polyclonal anti-human IgE FITC (green) to reveal IgE. Nuclei were counterstained with DAPI (blue) and samples analyzed by confocal microscopy. Data shown are representative of three independent experiments (scale bar is 50μm).

We further aimed to corroborate our results using confocal microscopy. Cells producing IgE, cells bearing IgE bound to CD23 and basophils as described above were transferred onto slides using a cytospin followed by staining with omalizumab-AF647, the respective isotype control (human IgG1κ-AF647) or polyclonal anti-human IgE FITC. Only IgE-producing cells were positive for omalizumab-AF647 but not the respective isotype control (**Figure 2A** and **Supplementary Figure S1**). No positive staining for omalizumab-AF647 was detected in cells bearing IgE bound to the CD23 receptor or FcϵRI. By contrast, polyclonal anti-human IgE stained IgE both in IgE-producing cells and cells bearing IgE in a receptor-bound form in all three cell types as shown in **Figure 2B**.

### Omalizumab Detects IgE-Producing Cells in Human Nasal Polyp Tissue

**Figure 3:**
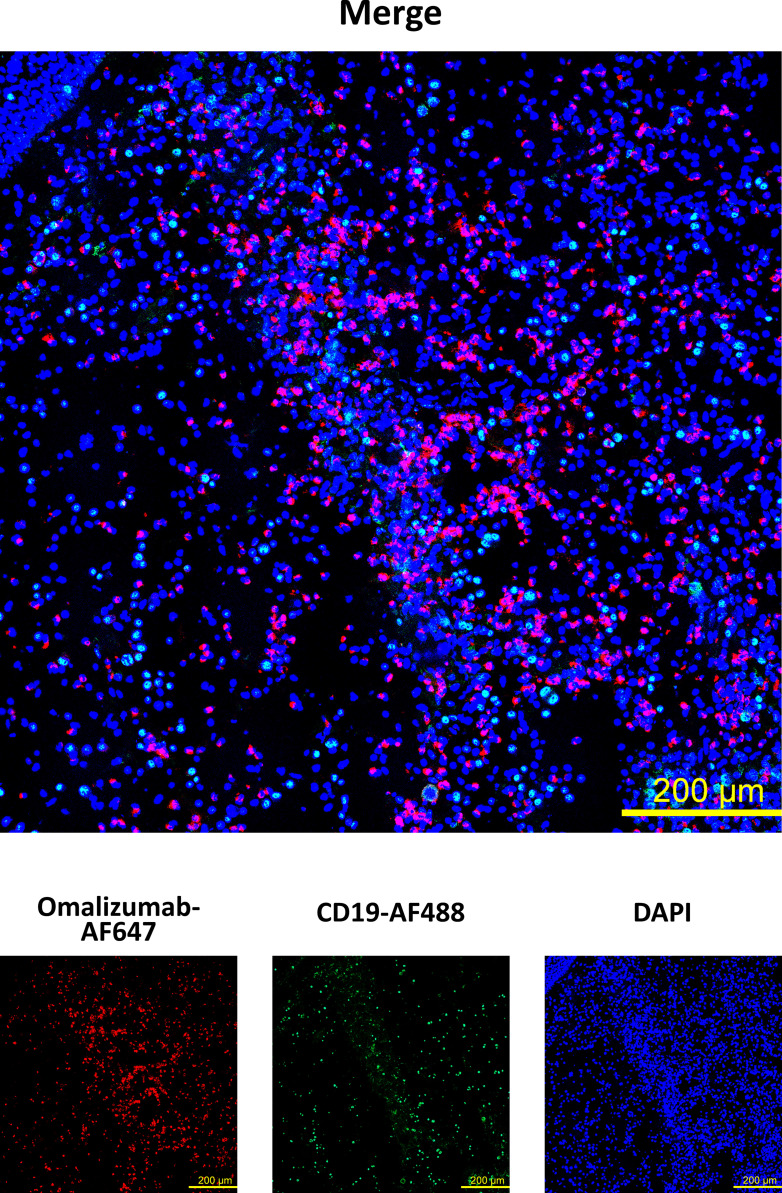
Detection of IgE-producing cells in a nasal biopsy by omalizumab using confocal microscopy. IgE producing cells (n=100,000, U266) were spiked onto cryosections from a nasal polyp using cytospin followed by staining with Alexa Fluor (AF) 647 labelled omalizumab (red) and anti-human CD19 (green). Nuclei were counterstained with DAPI (blue) and samples analyzed by confocal microscopy. Data shown are representative of three independent experiments (scale bar is 200μm).

Having established that omalizumab was able to detect cells bearing non-receptor-bound IgE in cell suspensions, we next assessed if it would detect these cells also in a tissue-based context using a nasal polyp biopsy. To that aim, we spiked IgE-producing cells (U266 cell line) onto polyp tissue sections prior to staining with omalizumab-AF647. Tissue samples were co-stained with anti-human CD19 to detect tissue-resident B cells and DAPI to stain for nuclei (**Supplementary Table S2**). We observed positive staining of IgE-bearing cells in human polyp tissue with omalizumab AF647 (**Figure 3**) while human IgG1k AF647 did not show any positive staining (**Supplementary Figure S2**).

### IgE-Producing Cells Present in PBMCs Are Revealed With a High Sensitivity by Omalizumab-Based Flow Cytometric Assays

**Figure 4:**
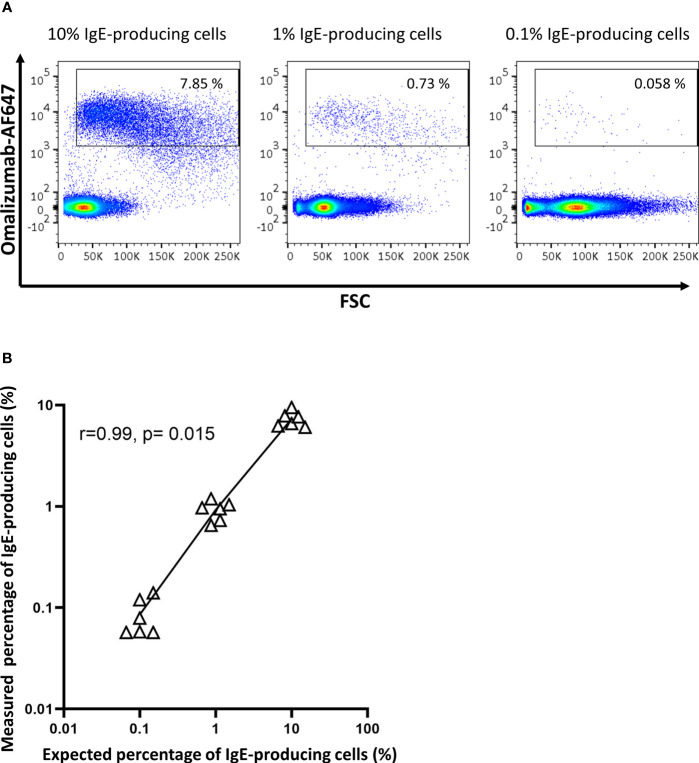
Omalizumab specifically detects IgE-producing cells with a high sensitivity. **(A)** 1 million peripheral blood mononuclear cells from a healthy donor were spiked with (left panel) 10% (n=100,000), (middle panel) 1% (n=10,000) or (right panel) 0.1% (n=1000) IgE-producing cells (U266) followed by detection with Alexa Fluor (AF) 647-labelled omalizumab using flow cytometry. Gates were set using isotype control for omalizumab-AF647. **(B)** Scatter plot of expected (x-axis, %) *versus* measured (y-axis, %) percentage of IgE-producing cells spiked into PBMCs. Black triangles represent individual replicates from experiments, line represents the linear regression slope. Pearson’s correlation coefficient with corresponding significance level is indicated in the figure. Data shown are representative of three independent experiments performed in duplicates.

To determine the sensitivity of omalizumab in detecting IgE BCR bearing cells within human PBMCs, we spiked the IgE-producing U266 cell line at different ratios into PBMCs of non-allergic patients. The ratio of U266:PBMCs ranged from 1:10 (10%) to 1:1000 (0.1%) prior to staining with omalizumab-AF647. Omalizumab-AF647-positive cells were observed in all three spiking conditions tested (10% spiking: 7.31 ± 1.18%, 1% spiking: 0.92 ± 0.18%, 0.1% spiking: 0.09 ± 0.03%). Representative plots from three independent experiments performed in duplicates are shown in **Figure 4A**. The measured percentage of IgE-producing cells in PBMCs by flow cytometry showed a strong and significant correlation with the expected percentage (Pearsons’s correlation coefficient r=0.99, p=0.015) (**Figure 4B**) and was highly reproducible (range of standard deviation: 0.03-1.18%).

### Detection of IgE BCR-Bearing B Cells Specific for the Major Birch Pollen Allergen Bet v 1 in Blood of Birch Pollen Allergic Patients by Omalizumab

**Figure 5:**
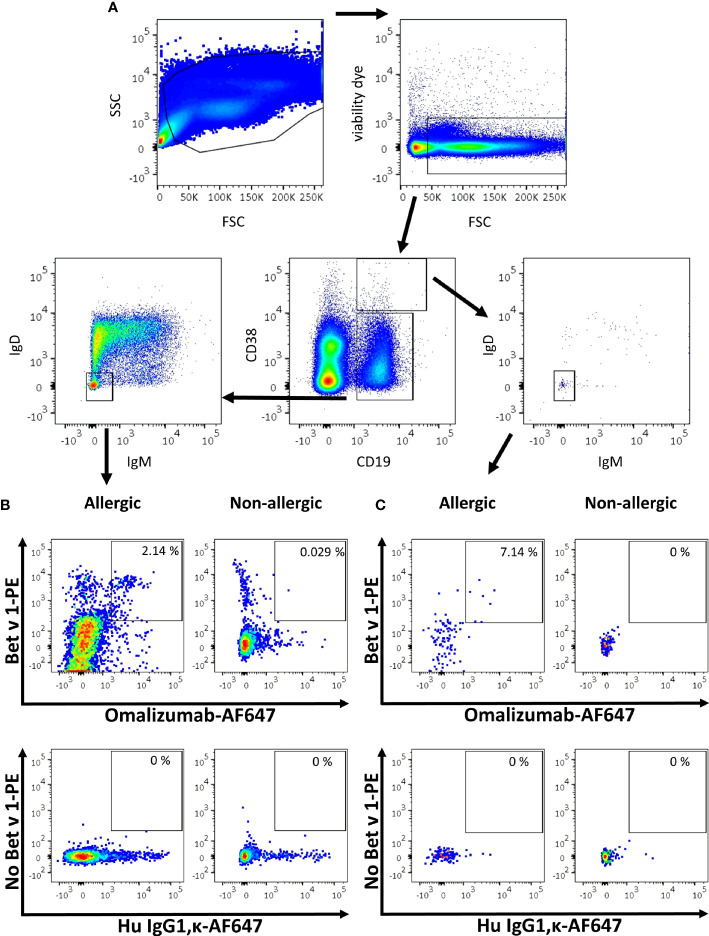
Gating strategy for the detection of IgE MBCs and PBs/PCs using omalizumab in allergic and non-allergic patients. **(A)** Overview of the sequential gating strategy for the detection of IgE positive B cells in an allergic subject. **(B, C)** Comparison of the percentage of IgE positive Bet v 1 positive **(B)** memory B cells or **(C)** plasma blasts/plasma cells in (left panels) an allergic subject previously exposed to birch pollen or (right panels) a non-allergic subject. Plots depict (upper panels) Alexa Fluor 647-labelled omalizumab (x-axis) against PE-labelled Bet v 1 (y-axis), and (lower panels) the respective isotype control (human IgG1, κ, x-axis) labelled with AF647 in the absence of Bet v 1 (y-axis). Plots are representative for 5 allergic and 3 non-allergic subjects.

As proof of concept, we aimed to identify blood-derived allergen-specific MBCs and/or PBs/PCs bearing a surface IgE BCR in birch pollen allergic patients by employing a sequential gating strategy based on recent publications (6, 7, 9). To that aim, PBMCs were isolated from birch pollen allergic donors one week after controlled nasal allergen challenge as well as from non-allergic donors. They were stained with fixable viability dye e780, anti-CD19 PerCP-Cy5.5, anti-CD38 BV510, anti-IgM BV421, anti-IgD BV605, Bet v 1 labelled with PE (Bet v 1-PE) and omalizumab-AF647. Gating was performed using corresponding isotype controls. After selecting for alive cells, we separated PBs/PCs from naïve and memory B cells based on their staining for CD19 and CD38 (**Figure 5A**). Subsequently we identified class-switched B cells by negative staining for IgM and IgD in both populations. In class-switched cells of the respective populations, we finally gated on cells double positive for the allergen Bet v 1-PE and omalizumab-AF647. Using this gating strategy, the percentage of Bet v 1^+^ IgE^+^ MBCs in the blood of allergen-exposed allergic patients ranged from 2.14% to 4.17% (mean=3.228 ± 0.721%, n=5) whereas they absent in non-allergic donors (mean=0.041 ± 0.003%, n=3) (**Figure 5B**) (**Supplementary Table S3**). We were also able to detect a small population of IgE^+^ PBs/PCs in 3 out of 5 allergic patients, the percentage of these cells ranged between 0% to 7.14% (mean=2.030 ± 2.655%, n=5) in the blood of birch pollen allergic patients one week after allergen contact and were completely absent in the case of non-allergic patients (**Figure 5C** and **Supplementary Table S3**).

## Discussion

Here we have shown that the anti-human IgE antibody omalizumab recognizes IgE B cell antigen receptor whilst omitting cells bearing IgE bound to CD23 or FcϵRI both in cellular suspension as well as in tissue using flow cytometry and confocal microscopy. Spiking experiments revealed that omalizumab was able to detect IgE-bearing cells over a wide range up to a ratio of 1 IgE-producing cells in 1 000 PBMCs thus proving its suitability for detection of rare IgE^+^ B cells in human samples. As a proof of concept, we developed a sequential gating strategy allowing for the unambiguous identification of allergen-specific IgE^+^ MBCs and PBs/PCs in allergic patients.

Detection of rare antigen specific cells is challenging. In this context, one important step consists of the optimization of the signal to noise ratio (35). One option is to reduce background noise by enriching for B cell subsets using e.g. magnetic separation or to exclude certain immune subsets in flow cytometric analysis using surface markers. With regards to IgE^+^ PCs, this strategy has successfully been applied in the past using microbeads containing antibodies against the plasma cell marker CD138 (36). However, it needs to be borne in mind with regards to IgE^+^ B cells which are very poorly characterized in terms of surface markers that this always implies the danger of accidentally deleting or missing cells of interest as CD138 is not expressed by all human plasma cells (37, 38). Thus, we chose an alternative approach which was to maximize the signal of the rare cells. To achieve this, we used an approach of double staining allergen-specific IgE^+^ B cells using both the allergen labelled with PE and omalizumab labelled with AF647 to clearly identify our population of interest. These two dyes have been carefully selected as they are currently among the brightest available fluorescent dyes, whereby especially AF647 has a very good profile in terms of photostability and signal to noise ratio as compared to other dyes in this channel such as APC (39, 40). Thus, we are able to maximize the signal and detect a clear Bet v 1 IgE double-positive population in allergic but in not non-allergic subjects without using amplification steps such as allergen biotin tetramers which may introduce further background noise due to detection with fluorescent streptavidin (41).

In our experiments in cell lines as well as in patient-derived PBMCs, omalizumab exclusively and sensitively detected IgE^+^ MBCs, confirming our previous preliminary results (20). Due to its binding region in Cϵ3 which is situated at the interface between the CD23 and FcϵRI binding site on IgE, this antibody was very well suited for our purposes. Additionally, we chose omalizumab as it is widely used and commercially available, however there are other conceivable alternative antibodies available potentially fulfilling these requirements. In this respect, Ligelizumab is also directed towards the Cϵ3 region of IgE and binds with 50 times higher affinity to IgE *in vitro* (42). However, one caveat regarding this antibody is that omalizumab and Ligelizumab have distinct binding sites and thus inhibition profiles: While Ligelizumab is superior to omalizumab in inhibiting IgE FcϵRI interaction, its ability to block binding of IgE to CD23 is reduced (43, 44). Moreover, Ligelizumab has even been shown to be able to bind to CD23-bound IgE in confocal stainings (44). Thus, its ability to distinguish between IgE B cell antigen receptor- and CD23-bound IgE is reduced. Another alternative approach is the usage of antibodies directed specifically to regions only expressed in the membrane but not secreted form of IgE such as the transmembrane or extracellular membrane-proximal domains (45). In this respect the monoclonal antibodies 4B12 binding to the proximal domain and the antibody 47HA directed towards the transmembrane M1 region have been shown to stain successfully free IgE (46, 47) but are so far not commercially available.

Our consecutive gating strategy was based on previously published approaches distinguishing between memory B cells and plasma cells/blasts based on the surface markers CD38 in conjunction with negative staining for IgM and IgD. In contrast to previous strategies, we did not further select for IgA and IgG negative cells for two reasons: Firstly, omalizumab is the first antibody used in flow cytometry that is able to directly identify B cells bearing membrane-bound IgE without need for additional selection steps. Secondly, B cells bear the Fcγ receptors family member FcγRIIB and thus gating for IgG negative cells may also exclude IgE^+^ B cells with Fcγ receptor bound IgG as commercial available anti-human IgG antibodies are not exclusively directed to free IgG present in the BCR (22–24). Alternatively, to using negative staining for IgM and IgD to select class switched B cells, CD27 in conjunction with CD19 and CD38 has been used as a marker to discriminate between naïve, memory cells and cells of the plasma cell lineage (8). However, it has been shown that CD27 is not expressed by all memory B cells but is rather an additional marker to discriminate memory B cell subsets (48–50). In this line using a stepwise exclusion approach by gating on CD19^+^CD38^dim^ cells followed by exclusion of IgM^+^IgD^+^IgG^+^IgA^+^ cells it has been demonstrated that IgE^+^ memory B cells expressing CD27 are most likely derived from a germinal center T cell-dependent pathway whereas IgE^+^ memory B cells lacking expression of CD27 most likely have a T cell-independent origin (7). Thus, as a substantial portion of IgE^+^ B cells do not express CD27, we did not use this marker for our gating approach.

Using a double staining with omalizumab and labelled birch pollen allergen, we identified an average of 3.228% IgE^+^ Bet v 1^+^ cells of total MBCs in 5 allergen-provoked birch pollen allergic patients, and 2.030% IgE^+^ Bet v 1^+^ cells of total PBs/PCs. When compared to the total number of B cells, these amounts correspond to 0.734% IgE^+^ Bet v 1^+^ MBCs of total CD19^+^ B cells (range: 0.4-1.1%) and 0.018% IgE^+^ Bet v 1^+^ PBs/PCs of total CD19+ B cells (range: 0.00-0.06%) (**Supplementary Table S3**). Bearing in mind that the patients in our study were exposed to allergen shortly before the blood draw and that we did not observe any allergen-specific IgE^+^ B cells in non-allergic patients or allergic patients prior to allergen challenge (data not shown), our data are in line with a recent study from R. Jimenez-Saiz suggesting that percentage of IgE^+^ memory cells in the absence of allergen challenge is magnitudes lower (6) than the previously reported 0.2% to 0.3% of total CD19^+^ B cells (7–9). Our data indicates that allergen-specific IgE^+^ memory B cells can be detected in blood but most likely only after allergen exposure, using a stringent gating approach. Where these cells may reside outside the season remains elusive (4). As the main aim of this study was the establishment of a consecutive gating strategy to reliably detect these cells, we applied our approach only in blood-derived cells. This is a limitation of this study as the majority of IgE-producing cells will reside elsewhere (4). However, based on our spiking experiments in nasal polyp tissue, we are confident that in future studies this strategy can also be used for the identification of IgE B cell antigen receptor-bearing and IgE-producing cells in various tissues.

In summary, we identified the anti-human IgE antibody omalizumab as a valuable tool for clear identification of IgE producing and IgE BCR-bearing B cells and established a step wise gating strategy allowing for the unambiguous identification of these cells in human blood derived PBMCs. Our strategy provides a useful tool for the investigation of human IgE memory cells.

## Data Availability Statement

The raw data supporting the conclusions of this article will be made available by the authors, without undue reservation.

## Ethics Statement

The studies involving human participants were reviewed and approved by Ethical Committee of the Medical University of Vienna. The patients/participants provided their written informed consent to participate in this study.

## Author Contributions

MZ, JE-D, and RV designed the study and wrote the manuscript. HB, SF, VN-L, MB, NC, VS, AT, and SV-M provided reagents and participated in the design and/or conduct of experiments. MZ performed the experiments and wrote the first draft of the manuscript. All co-authors critically revised the manuscript. All authors contributed to the article and approved the submitted version.

## Funding

This work was supported by the Country of Lower Austria’s funded Danube Allergy research cluster, by grants DK W 1248-B30 and SFB F4613 from the Austrian Science Fund (FWF), by a Megagrant of the Government of the Russian Federation, grant No 14.W03.31.0024 and in part by the Russian Science Foundation, grant No 21-15-00286.

## Conflict of Interest

RV has received research grants from Worg Pharmaceuticals, Hangzhou, China, HVD Biotech, Vienna, Austria and Viravaxx, Vienna, Austria. He serves as a consultant for Viravaxx and Worg.

The remaining authors declare that the research was conducted in the absence of any commercial or financial relationships that could be construed as a potential conflict of interest.

## Publisher’s Note

All claims expressed in this article are solely those of the authors and do not necessarily represent those of their affiliated organizations, or those of the publisher, the editors and the reviewers. Any product that may be evaluated in this article, or claim that may be made by its manufacturer, is not guaranteed or endorsed by the publisher.
