# Supplementary material for: Tracing Human IgE B Cell Antigen Receptor-Bearing Cells With a Monoclonal Anti-Human IgE Antibody That Specifically Recognizes Non-Receptor-Bound IgE
Source: Front Immunol. 2021 Dec 20;12:803236. doi: 10.3389/fimmu.2021.803236 (PMC8721004; doi:10.3389/fimmu.2021.803236)
Supplement: Supplementary file 1 [file DataSheet_1.docx]

Supplementary Material

Tracing Human IgE B Cell Antigen Receptor-Bearing Cells with a monoclonal anti-human IgE antibody that specifically recognizes non-receptor-bound IgE

Mohammed Zghaebi^1^ (ORCID: 0000-0002-7636-2601), Maria Byazrova^2,3^(ORCID: 0000-0002-9858-7596), Sabine Flicker^4^ (ORCID: 0000-0003-4768-8693), Sergio Villazala-Merino^1^ (ORCID: 0000-0002-6791-3272), Nicholas J. Campion^1^ (ORCID: 0000-0003-3345-9486)_,_ Victoria Stanek^1^ (ORCID: 0000-0003-3445-0934)_,_ Aldine Tu^1^, Heimo Breiteneder^5^ (ORCID: 0000-0003-2022-8689), Alexander Filatov^2,3^ (ORCID: 0000-0002-6460-9427), Musa Khaitov^2,6^ (ORCID: 0000-0003-4961-9640), Verena Niederberger-Leppin^1^ (ORCID: 0000-0002-2918-2608)_,_ Julia Eckl-Dorna^1*^(ORCID: 0000-0001-5981-1607), Rudolf Valenta^2,5.7,8^(ORCID: 0000-0001-5944-3365)

^1^ Department of Otorhinolaryngology, Medical University of Vienna, Vienna, Austria

^2^ National Research Centre (NRC) Institute of Immunology, Federal Medical-Biological Agency (FMBA) of Russia, Moscow, Russia.

^3^ Department of Immunology, Faculty of Biology, Lomonosov Moscow State University, Moscow, Russia

^4^ Division of Immunopathology, Department of Pathophysiology and Allergy Research, Center for Pathophysiology, Infectiology and Immunology, Medical University of Vienna, Vienna, Austria

^5^ Division of Medical Biotechnology, Department of Pathophysiology and Allergy Research, Center for Pathophysiology, Infectiology and Immunology, Medical University of Vienna, Vienna, Austria

^6^ Pirogov Russian National Research Medical University, Moscow, Russia

^7^ Department of Clinical Immunology and Allergy, Sechenov First Moscow State Medical University, Moscow, Russian Federation

^8^ Karl Landsteiner University of Health Sciences, Krems, Austria

# Supplementary Figures and Tables

## Supplementary Figures


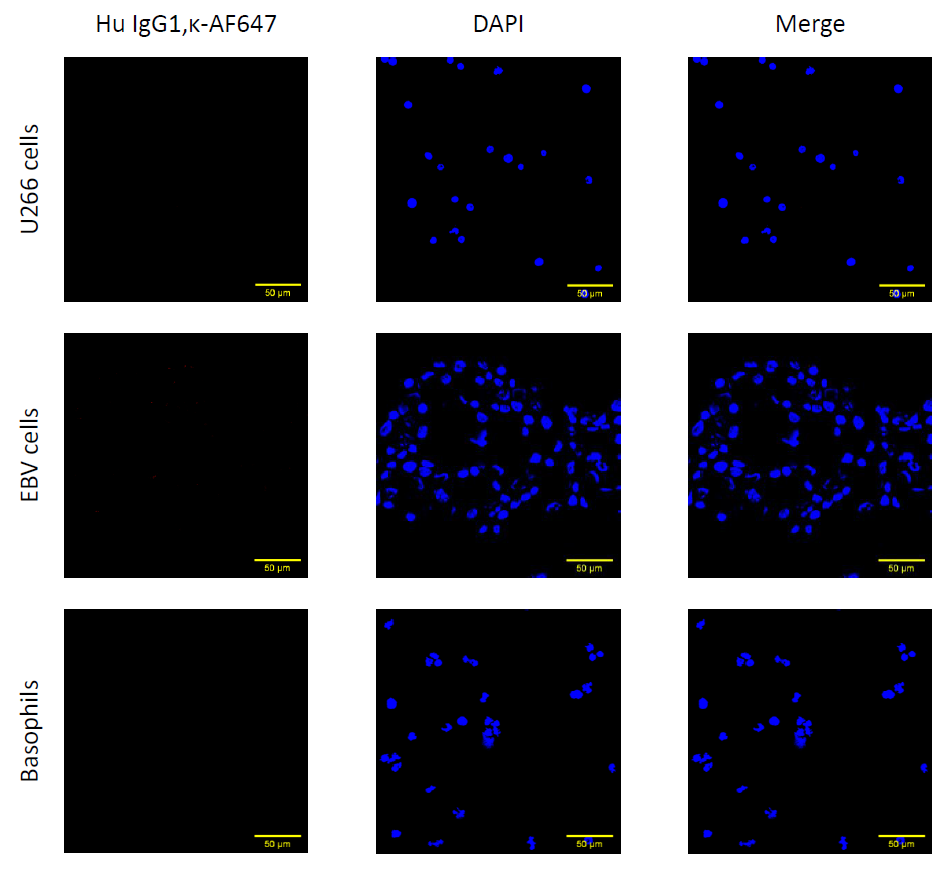


**Supplementary figure 1: Isotype controls for confocal imaging of IgE-producing cells or cells bearing receptor-bound IgE by omalizumab or polyclonal anti-human IgE. (A-B)** Representative confocal images of **(upper panels)** U266, IgE-producing cell line **(middle panels)** an Epstein Barr virus-transformed human cell line bearing IgE-bound to the low affinity receptor for IgE, CD23 and **(lower panels)** basophils enriched from human blood and bearing IgE bound to the high affinity receptor for IgE, FcεRI. Cells were stained with Alexa Fluor (AF) 647 labelled Human IgG1,κ. Nuclei were counterstained with DAPI (blue) and samples analysed by confocal microscopy. Data shown are representative of three independent experiments (scale bar is 50μm).


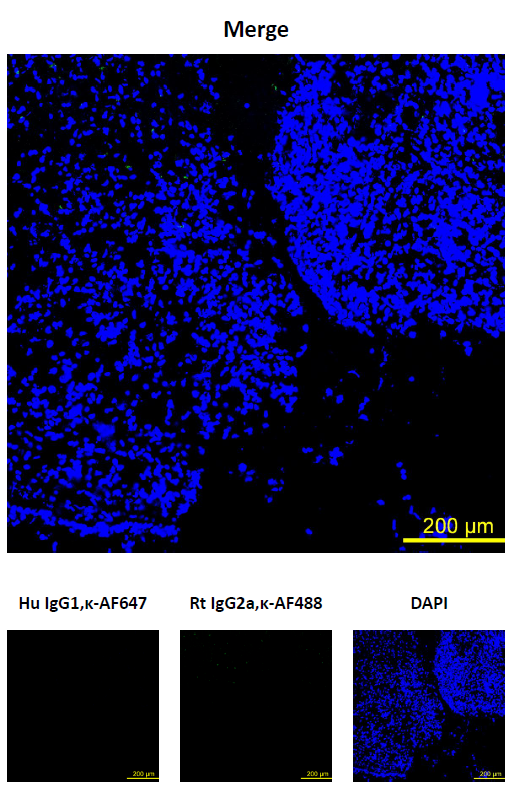


**Supplementary figure 2:** **Isotype controls for detection of IgE-producing cells in a nasal biopsy by omalizumab using confocal microscopy.** IgE-producing cells (n=100,000, U266) were spiked onto cryosections from a nasal polyp using cytospin followed by staining with AF647 labelled human (Hu) IgG1,κ (red) and rat (Rt) IgG2a,κ-AF488 (green). Nuclei were counterstained with DAPI (blue) and samples analysed by confocal microscopy. Data shown are representative of three independent experiments (scale bar is 200μm).

## Supplementary Tables

**Table S1: Antibodies used in flow cytometry experiments.**

| **Antibody** | **Conjugate** | **Company** | **Clone** | **CAT No.** |
| --- | --- | --- | --- | --- |
| Anti-human CD19 | PerCP-CY5.5 | BD Biosciences | HIB19 | 561295 |
| Anti-human CD38 | BV510 | BD Biosciences | HIT2 | 563251 |
| Mouse IgG1,κ isotype control | BV510 | BD Biosciences | X40 | 562946 |
| Mouse anti human IgG | PE-CY7 | BD Biosciences | G18-1453 | 561298 |
| Mouse IgG1,κ isotype control | PE-CY7 | BD Biosciences | MOPC-21 | 565573 |
| Anti-human IgD | BV605 | BD Biosciences | IA6-2 | 563313 |
| IgG2a,κ Isotype control | BV605 | BD Biosciences | G155-178 | 562778 |
| Anti-human IgM | BV421 | BD Biosciences | G20-127 | 562618 |
| Mouse IgG1,κ Isotype control | BV421 | BD Biosciences | X40 | 562438 |
| Anti-human CD23 | PE | ThermoFisher Scientific | EBVCS2 | 12-0238-42 |
| Mouse IgG1,κ Isotype control | PE | ThermoFisher Scientific | P3.6.2.8.1 | 12-4714-82 |
| Anti-human CD123 | BV421 | Biolegend | 6H6 | 306018 |
| Mouse IgG1,κ Isotype control | BV421 | Biolegend | MOPC-21 | 400158 |
| Anti-human FcεRI | PE | ThermoFisher Scientific | AER-37 | 12-5899-42 |
| Mouse IgG2b,κ Isotype Control | PE | ThermoFisher Scientific | eBMG2b | 12-4732-81 |
| Fixable viability dye | eFluor 780 | ThermoFisher Scientific |  | 65-0865-14 |

**Table S2: Antibodies used in immunofluorescence experiments.**

| **Antibody** | **Conjugate** | **Company** | **Clone** | **CAT No.** |
| --- | --- | --- | --- | --- |
| Anti-human IgE | FITC | SeraCare |  | 02-10-04 |
| Anti CD19 | No conjugate | ThermoFisher Scientific | 6OMP31 | 14-0194-82 |
| Rat IgG2a,κ isotype control | No conjugate | ThermoFisher Scientific | eBR2a | 14-4321-82 |
| Goat anti-rat IgG (H+L) | Alexa Fluor 488 | ThermoFisher Scientific |  | A11006 |
| DAPI |  | ThermoFisher Scientific |  | D1306 |

**Table S3: Percentages of IgE^+^ Bet v1^+^ MBCs and PBs/PCs in allergic patient after allergen contact.**

|  | Donor ID | Birch pollen allergy | Percentage of events in the MBCs IgE Bet v 1 gate (%) | Percentage of IgE^+^ Bet v 1^+^ MBCs of total B cells (%) | Percentage of events in the PBs/PCs IgE Bet v 1 gate (%) | Percentage of IgE^+^ Bet V1^+^ PCs/PBs of total B cells (%) |
| --- | --- | --- | --- | --- | --- | --- |
|  | 001 | Yes | 2.14 | 0.41 | 7.14 | 0.02 |
|  | 002 | Yes | 2.7 | 0.54 | 0 | 0 |
|  | 003 | Yes | 3.68 | 1.10 | 1.93 | 0.06 |
|  | 004 | Yes | 3.45 | 0.92 | 1.08 | 0.01 |
|  | 005 | Yes | 4.17 | 0.70 | 0 | 0 |
| Average |  |  | 3.228 | 0.734 | 2.030 | 0.018 |
| Standard deviation |  |  | 0.721 | 0.249 | 2.655 | 0.022 |
